# Pancreatic Islet Survival and Engraftment Is Promoted by Culture on Functionalized Spider Silk Matrices

**DOI:** 10.1371/journal.pone.0130169

**Published:** 2015-06-19

**Authors:** Ulrika Johansson, Massimiliano Ria, Karin Åvall, Nancy Dekki Shalaly, Sergei V. Zaitsev, Per-Olof Berggren, My Hedhammar

**Affiliations:** 1 Department of Anatomy, Physiology and Biochemistry, Swedish University of Agricultural Sciences, the Biomedical Centre, S-750 07, Uppsala, Sweden; 2 The Rolf Luft Research Center for Diabetes and Endocrinology, Karolinska Institutet, Karolinska University Hospital, S-171 76 Stockholm, Sweden; 3 Division of Protein Technology, School of Biotechnology, KTH Royal Institute of Technology, S-106 91 Stockholm, Sweden; Broad Institute of Harvard and MIT, UNITED STATES

## Abstract

Transplantation of pancreatic islets is one approach for treatment of diabetes, however, hampered by the low availability of viable islets. Islet isolation leads to disruption of the environment surrounding the endocrine cells, which contributes to eventual cell death. The reestablishment of this environment is vital, why we herein investigated the possibility of using recombinant spider silk to support islets *in vitro* after isolation. The spider silk protein 4RepCT was formulated into three different formats; 2D-film, fiber mesh and 3D-foam, in order to provide a matrix that can give the islets physical support *in vitro*. Moreover, cell-binding motifs from laminin were incorporated into the silk protein in order to create matrices that mimic the natural cell environment. Pancreatic mouse islets were thoroughly analyzed for adherence, necrosis and function after *in vitro* maintenance on the silk matrices. To investigate their suitability for transplantation, we utilized an eye model which allows *in vivo* imaging of engraftment. Interestingly, islets that had been maintained on silk foam during *in vitro* culture showed improved revascularization. This coincided with the observation of preserved islet architecture with endothelial cells present after *in vitro* culture on silk foam. Selected matrices were further evaluated for long-term preservation of human islets. Matrices with the cell-binding motif RGD improved human islet maintenance (from 36% to 79%) with preserved islets architecture and function for over 3 months *in vitro*. The islets established cell-matrix contacts and formed vessel-like structures along the silk. Moreover, RGD matrices promoted formation of new, insulin-positive islet-like clusters that were connected to the original islets via endothelial cells. On silk matrices with islets from younger donors (<35 year), the amount of newly formed islet-like clusters found after 1 month in culture were almost double compared to the initial number of islets added.

## Introduction

Transplantation of pancreatic islets has the potential to become a widely applicable treatment for insulin-dependent diabetes, but unfortunately currently available procedures suffer from low efficacy and limited reproducibility [1, 2]. The low success rates are incompletely understood but major factors are qualitative and quantitative deficiencies of the transplanted islet cells and gradual loss of function [2, 3]. Only half of the embolized islets have been detected in the liver immediately after transplantation via the portal vein, indicating that the islets are lost by rapid processes such as instant blood-mediated inflammatory reactions and/or hemodynamic shear stress [4, 5]. Usually islets from two to four donors are required for each recipient to accomplish successful transplantation [6]. Both freshly isolated and cultured islets have been used in experimental and clinical transplantations [7]. Conventionally, islets are cultured while free floating in medium in non-adherent plastic culture wares, although the islets in their natural niche are surrounded by extracellular matrix (ECM) that gives both mechanical support and biochemical interactions. The ECM is destroyed during islet isolation, which has been suggested as a major cause of the limited survival of functional beta cells [8–10]. A temporary, artificial ECM-like support could potentially compensate for the partially destroyed peri-vascular and peri-insular basement membranes and allow both restoration and maintenance of functional islets after isolation. First, a support that increases the *in vitro* viability of islets would facilitate logistics around transplantation, simply by maintaining more islets viable for longer times before transplantation. Secondly, a support that allows *in vitro* establishment of viable islets, with cell-matrix contacts and vascular network, would enable relevant *in vitro* studies of the biological mechanisms behind diabetes. Thirdly, the establishment of an environment optimized for islet cells could allow the design of an artificial, islet-carrier, to support and protect the islets also during and after transplantation. However, in order to develop a functional, ECM-like support for islets cells, a highly versatile biomaterial is needed.

Recombinant spider silk is a promising biomaterial, especially for biomedical applications, due to favorable properties from a biocompatibility point of view, *i*.*e*. low immunogenic response when introduced *in vivo* [11, 12] and surface properties governing cell adhesion [13–15]. 3D scaffolds have been shown to enhance survival, growth and function of cells compared to when cultured on 2D culture dishes [16]. The highly polymeric nature of spider silk proteins allows processing into various formats, including 3D foam [15] that can be optimized to match the sizes of pancreatic islets. The ECM support that naturally surrounds pancreatic islet cells is provided by local endothelial cells [17] and is composed of collagen IV, laminin (mainly 511, 411 and 111 [18]) and fibronectin [19]. In recent years, *in vitro* islet research has produced extensive evidence that cell-matrix interactions through cell binding motifs in these proteins contribute to improved islet cell survival and function [20]. By the usage of specifically functionalized spider silk proteins as building blocks for fiber formation, we can further optimize the matrices with cell binding motifs to imitate the natural ECM support [15].

The aim of our study was to establish new methodologies for long-term *in vitro* maintenance of viable pancreatic islets prior to transplantation. Matrices of recombinant spider silk were developed and used in different formats and with different incorporated peptide motifs to evaluate their effect on islet adherence, function and survival both *in vitro* and *in vivo*. The long-term goal is to accomplish pre-requisites for transplantable, functional pancreatic islets.

## Materials and Methods

### Production of recombinant spider silk

Conventional molecular cloning techniques were used to insert short peptides (RGD, RGE, IKVAV and YIGSR) at the N-terminus of the recombinant spider silk protein 4repCT [21]. Briefly, the 4repCT sequence was amplified by PCR using sense primers including bases corresponding to respective peptide. The resulted products were cleaved using *Eco*RI and *Hind*III before ligation into the pHisTrxHis4repCT vector, cleaved with the same restriction enzymes. The different pHisTrxHis4repCT constructs were then used to transform *Escherichia coli* BL21 (DE3) cells (Merck Biosciences). Corresponding proteins were expressed and recovered as previously described [21]. A procedure for depletion of Lipopolysaccharides (LPS) was also included [13] using EndoTrap columns (Profos AG). After purification, the protein solution was sterile filtered (0.22 μm) and concentrated to 1 or 3mg/mL by centrifugal filtration (Amicon Ultra, Millipore). The proteins were coated in 24-well plates for suspension cultures (Sarstedt) in three different formats *i*.*e*. fiber, film and foam as described in Widhe *et al* [15]. Briefly, films and foams were made by casting of a protein solution as such or foamed, and allowed to incubate over night at 20–30°C. Fibers were made by gentle wagging of a protein solution over night at room temperature. The thereafter formed fiber bundles were chopped into pieces and allowed to dry onto a cell culture well.

### Pancreatic islet donors and islet isolation

As rodent pancreatic islet donors 37 C57Bl/6J mice were used for *in vitro* islet studies. Typically, each experiment required islets from 1–2 mice, and the same islets were used for several types of analysis (*e*.*g*. adhesion, viability and function). All experiments were repeated at several occasions, denoted *n* in the figure legends. All animal work within this study was conducted in accordance with the Swedish Animal Research Committee’s guidelines. The study was approved by the Swedish Animal Research Committees at Karolinska Institutet, Stockholm, Sweden (permit number N185/10). The mice were sacrificed by CO_2_ asphyxiation followed by cervical dislocation. Collagenase (1 mg mL^-1^) in Hank’s Balanced Salt Solution (HBSS) was injected into the pancreatic duct to induce digestion. The pancreas was placed in glass vials in 37°C shaking water bath for 20 minutes before transferred into polystyrol tubes where sedimentation occurred for 5 minutes. The supernatant was removed and the remaining pellet washed twice in HBSS containing HEPES (25 mM) and BSA (1 mg mL^-1^). The islets were handpicked under a stereomicroscope into a Petri dish with complete RPMI 1640 medium containing L-glutamine (2 mM), penicillin (100 U mL^-1^), streptomycin (100 ug mL^-1^), 10% heat-inactivated fetal bovine serum (FBS) and glucose (11 mM).

Human islets from declared diseased donors (n = 10) were provided by the Nordic Islet Transplantation Program (www.nordicislets.org), who use a modified [22] semi-automated digestion-filtration method [23] for islet isolation. Islets were released for research after approval by the ethics committee at Uppsala University Hospital. The human islets were obtained from the unavoidable excess of islets generated within the Nordic Network for Clinical Islet Transplantation. Only organ donors who explicitly had agreed to donate for scientific purposes were included. Informed written consent to donate organs for medical and research purposes was obtained from donors, or relatives of donors, by the National Board of Health and Welfare (Socialstyrelsen), Sweden. The experimental procedures performed on the human islets were approved Swedish Animal Research Committees at Karolinska Institutet, Stockholm, Sweden (permit number 2011/146732). The human islets were cultured in CMRL-1066 (ICN Biomedicals) supplemented with HEPES (10 mM), L-glutamine (2 mM), Gentamycin (50 mg ml^-1^), Fungizone (0.25 mg ml^-1^, Gibco), Ciprofloxacin (20 mg ml^-1^, Bayer Healthcare AG), nicotinamide (10 mM), and 10% FBS.

### *In vitro* evaluation of pancreatic islets on silk

#### Culture and analysis of islet adherence

The matrices (film, fiber or foam) were washed twice in PBS and incubated in complete RPMI 1640 medium (mice islets) or complete CMRL-1066 (human islets) for 30 minutes. Islets were thereafter handpicked and placed on top of the matrices to enable adherence. As controls, islets were cultured in suspension with the same complete medium (as described above) in wells without coating. For each well, 10 freshly isolated mice islets in duplicates, or 20 human islets in triplicates, were plated for each variant of silk.

The plates were placed in an incubator (37°C, 5% CO_2_). Mice islets were cultured for 2 weeks and the human islets for up to 3 months. The islets were allowed to adhere to the matrices and the number of adhered islets was counted each day until day 5, and at the endpoint. The medium was changed at day 2, 5 and thereafter once every week. Glucose stimulation of insulin release was performed on day 2 and day 5, and, if long-termed culture, after 1, 2 and 3 months.

#### Insulin release

For initial experiments, glucose stimulation was performed in wells containing both adhered and non-adhered islets. In order to analyze adhered and non-adhered islets separately, the non-adhered islets were put in new, empty wells during the insulin release procedure. The islets were washed twice with PBS and thereafter pre-incubated for 30 minutes in a basal buffer containing NaCl (125 mM), KCl (5.9 mM), MgCl_2_ (1.2 mM), CaCl_2_ (1.28 mM) and HEPES (25 mM, pH 7.4), BSA (1 mg mL^-1^) and glucose (3 mM). The basal buffer was discarded and replaced with new basal buffer and further incubated for 30 minutes before collected. Then, the same buffer, but with a stimulatory concentration of glucose (16.7 mM) was added and incubated for 30 minutes before collected. All samples were stored at -20°C until analyzed. Thereafter the non-adhered islets were put back to wells with matrices.

#### Insulin measurement

The released insulin was analyzed with ELISA kit (Mercodia). All samples were diluted according to instructions from the supplier, in order to fit the supplemented standard curve. As the number of islets differed, values were also calculated as ng (10 islets*hour)^-1^. Stimulation index was calculated for each well separately.

#### Intracellular calcium

Islet function was also investigated by measurements of changes in [Ca^2+^]_i_ during stimulation with high glucose (11 mM) or depolarization with KCl (25 mM) as previously described [24, 25]. Fura-2/AM (Molecular Probes) was used to indicate [Ca^2+^]_i_. After 1–2 days on WT or RGD silk, or in control wells, islets (n = 17–28) were loaded with Fura-2/AM (2 μM) in a basal buffer containing NaCl (125 mM), KCl (5.9 mM), CaCl_2_ (1.3 mM), MgCl_2_ (1.2 mM), and HEPES (25 mM,pH 7.4), holding a glucose concentration of 3 mM for 50 minutes. Thereafter the islets were attached with BD PuraMatrix Peptide (BD Pharmingen) to a cover slip suited for a custom-built open perifusion chamber (volume of 150 μL). The chamber was placed in an analyzing microscope (Zeiss Axiovert 135) connected to a Spex Fluorolog spectrophotometer and maintained at 37°C. [Ca^2+^]_i_ was measured by Fura-2/AM at 340/380-nm fluorescence ratio during perifusion of the islet by the following protocol; 1) Basal glucose (3 mM) for 50 seconds, 2) stimulation with glucose (11 mM) for 950 seconds, 3) return to basal level of glucose (3 mM) for 500 seconds, and 4) depolarisation with KCl (25 mM) for 250 seconds. Calculations were done on responding islets as previously described [26].

#### Morphometry

Inverted light microscopy (Nikon Eclipse Ti equipped with differential interference contrast) was used to monitor islets during culture and at the endpoint. Live microscopy as well as captured micrographs were used in order to monitor and analyze changes in the islet morphology *e*.*g*. islet integrity, necrosis, cell outgrowth and islet-like clusters.

Freshly isolated islets typically have a three-dimensional architecture which is disrupted if islets viability is compromised. Maintenance of islets during the culture period was determined by comparing the amounts of remaining islets which still had intact islet architecture to the amount of islets added at the starting point.

Isolated islets are avascular and rely upon diffusion for nutrition and oxygenation in culture, and therefore suffer from ischemia which in turn results in central islet necrosis. During live bright field light microscopy of islets, the necrotic areas can be seen as dark spots, called necrotic bodies, and the amount of these entities were counted per islet. Due to the three-dimensional architecture of the islets, it is difficult to visualize the necrotic status from captured micrographs, why representative micrographs of islets with calculated necrotic bodies (indicated as number) are shown in S1 Fig.

At the end point, fixated and stained samples were used to measure cell outgrowth by calculating the cell outgrowth emanating from the islet either as sprouts (CD31 positive) or as cell outgrowth (CD31 negative).

Newly formed islet-like clusters were calculated in each well with human islets. The islet-like clusters differed in morphology, with smaller size and form, compared to the denser and more round islets.

#### Immunohistochemical analysis

At the endpoint of *in vitro* culture, mouse and human islets were fixed in 4% paraformaldehyde for 15–30 minutes, washed in PBS and directly stained (mouse islets) or incubated in 20% sucrose in PBS (human islets) overnight at 4°C. Thereafter they were permeabilized in 0.3% Triton-X/PBS for 15–20 minutes and treated with either 6% fetal calf serum (mouse islets) for 1hour or 10% goat serum (human islets) for 30 minutes at RT. Immunohistochemical and immunocytochemical stainings were performed using guinea pig anti-mouse insulin (1:100, AbCam) or guinea pig anti human insulin (1:100, DAKO) followed by Alexa Flour 488-labeled goat anti-guinea pig (1:1000, Invitrogen), and rabbit anti mouse CD31 (1:50, AbCam) or mouse anti-human CD31 (1:100, BD Pharmingen) followed by Alexa flour 594-labeled goat anti-rabbit (1:1000) or Alexa Flour 545-labeled rabbit anti-mouse (1:1000), overnight at 4°C and 1h at RT, respectively. Counterstaining was performed with DAPI (1:1000; Sigma-Aldrich). The same protocol using the endothelial cell marker CD31 was also used for analysis of intra islets endothelial cells content in mouse islets directly after isolation and after 2 weeks of culture, as well as for analysis of cell growth emanating from the human islets after long term culture.

### *In vivo* evaluation using the mouse eye

#### Transplantation into the anterior chamber of an eye

All transplantations were done using essentially the same procedure [27]. C57BL/6 mice were anesthetized with 2% isoflurane (vol/vol). Eyes were kept humidified (ophthalmologic eye drops) to avoid drying of the cornea. Under a stereomicroscope, the cornea was punctured close to the sclera at the bottom part of the eye with a 25-gauge insulin needle. Using the needle, we made a small radial incision of approximately the size of the eye cannula (0.44 mm). For this incision, the needle was barely introduced into the anterior chamber, thus avoiding damage to the iris and bleeding. The blunt eye cannula was then gently inserted through this incision, first perpendicular to the surface of the cornea and then parallel to the cornea. When the cannula had been stably inserted into the anterior chamber, the transplant was slowly injected in the smallest volume possible of sterile saline solution into the anterior chamber, where it settled on the iris. After injection, the cannula was carefully and slowly withdrawn to hinder the transplant from flowing back through the incision. After awakening, the mice were put back in the cages and monitored until full recovery, and observed regularly thereafter until endpoint. Analgesia was obtained after surgical procedures with buprenorphine (0.05–0.1 mg/kg s.c.).

First, foam matrices of WT and RGD (n = 3) were transplanted without islets in order to investigate the *in vivo* compatibility of the silk material itself. The animals were followed for signs of illness and eye inflammation. Micrographs of the transplanted foam matrices were taken regularly up to the endpoint (1 year) in order to monitor signs of degradation.

Thereafter, islets that had been maintained for 2 weeks *in vitro* with or without RGD matrix were transplanted (3 experiments, 4–5 animals per experiment, 5–7 islets per eye). Islets were dissected out together with the nearest surrounding matrices (RGD) or transferred from culture media (free floating control) to sterile PBS solution and aspirated into a 27-gauge eye cannula, prepared by adapting a blunt ended patch clamp glass capillary, connected to a 1-mL Hamilton syringe (Hamilton) via 0.4-mm polyethylene tubing (Portex) and thereafter transplanted.

#### Imaging of transplanted matrices and/or islets within the mouse eye

*In vivo* imaging of foam and/or islets in the anterior chamber of the eye of the transplanted animals, was performed as previously reported [27]. Briefly, mice were anesthetized with 2% isoflurane (Baxter) air mixture and placed on a heating pad, and the head was restrained with a head holder. The eyelid was carefully pulled back and the eye gently supported. For imaging, an upright laser scanning confocal microscope based on a Leica TCS-SP5 II (Leica Microsystems) was used together with long-distance water-dipping objectives (Leica HXC-APO 10x/0.3, 20x/0.5, and 40x/0.8 NA). Viscotears (Novartis) was used as an immersion liquid between the eye and the objective. Imaging of islet morphology was done by laser illumination at 633 nm and reflected light was collected between 630 and 637 nm. Scanning speed and laser intensities were adjusted to avoid cellular damage to the mouse eye, matrix or the islet graft. Pictures of vascularization were obtained by acquiring a set of z-stack frames through the whole islet with both laser at 633 nm and using a digital microscope color camera (DFC295, Leica Microsystems) to collect real-time images of vasculature. Post-processing, analysis, visualization and measurement of *in vivo* transplanted islets were performed with Volocity (PerkinElmer) software, to enable evaluation of size and vasculature of the islets *in vivo*.

#### Immunohistochemical analysis of explants

At the endpoint, the eyes with the transplants were removed from terminated animals and fixed in 4% paraformaldehyde for 24 hours then kept in 70% ethanol until they were paraffin-embedded and sliced to 5 μm thick sections. After deparaffinization slides were blocked with 5% Normal Goat Serum in PBS for 1.5 h at RT and stained for insulin as described above. Slides were imaged and screened for islets, using the same equipment and parameters as described for *in vivo* imaging. Alexa Flour 488 was excited at 488 nm and emission light was collected between 500 and 550 nm, while Alexa Flour 594 was excited at 594 nm and emission light was collected between 620 and 700 nm. For hematoxylin and eosin (H/E) staining, every 10^th^ slide carrying 3–4 consecutive sections was subjected to a standard regressive hematoxylin protocol and counterstained in eosin. Briefly, after deparaffinization and rehydration slides were stained in freshly filtered Accustain Harris Hematoxylin Solution (Sigma-Aldrich), differentiated in 0.25% acid alcohol solution, blued in 0.1% sodium bicarbonate and counterstained in Eosin Y aqueous Solution (Sigma-Aldrich); slides were rinsed in running tap water between each step. Finally slides were dehydrated through graded alcohols to xylene and mounted in Pertex organic mounting medium (Histolab, Gothenburg, Sweden).

Cell death and intra islet vasculature were histologically evaluated in each islet, typically found in several consecutive H/E stained eye sections. Cell death was graded by looking at following criteria; nuclei size, nuclei fragmentation, nuclei lysis and complete loss of nuclei. Each islet was graded 0–5, where 0 means no visual cell death and 5 means high degree of cell death. The intra islet vasculature was screened for erythrocyte content and evaluated as percentage of islets with erythrocytes found within the vasculature per eye. Some sections were stained for insulin (as described above) and areas within islets that were both negative for insulin and not corresponding to vasculature were graded 0–5, where 0 means no insulin negative endocrine cells and 5 means high degree of insulin loss.

### Statistical analysis

Data analysis was performed using the statistical program Prism 5 (Graph pad). Results are expressed as mean ± SEM and evaluated using paired Student’s t-test, two-tailed Wilcoxon test for paired data and Two-way ANOVA with Bonferroni correction as post hoc test, with significance set at *p≤0.*05*, **p≤0.01 and ***p≤0.001.

## Results

### Recombinant spider silk functionalized to support pancreatic islets

**Fig 1:**
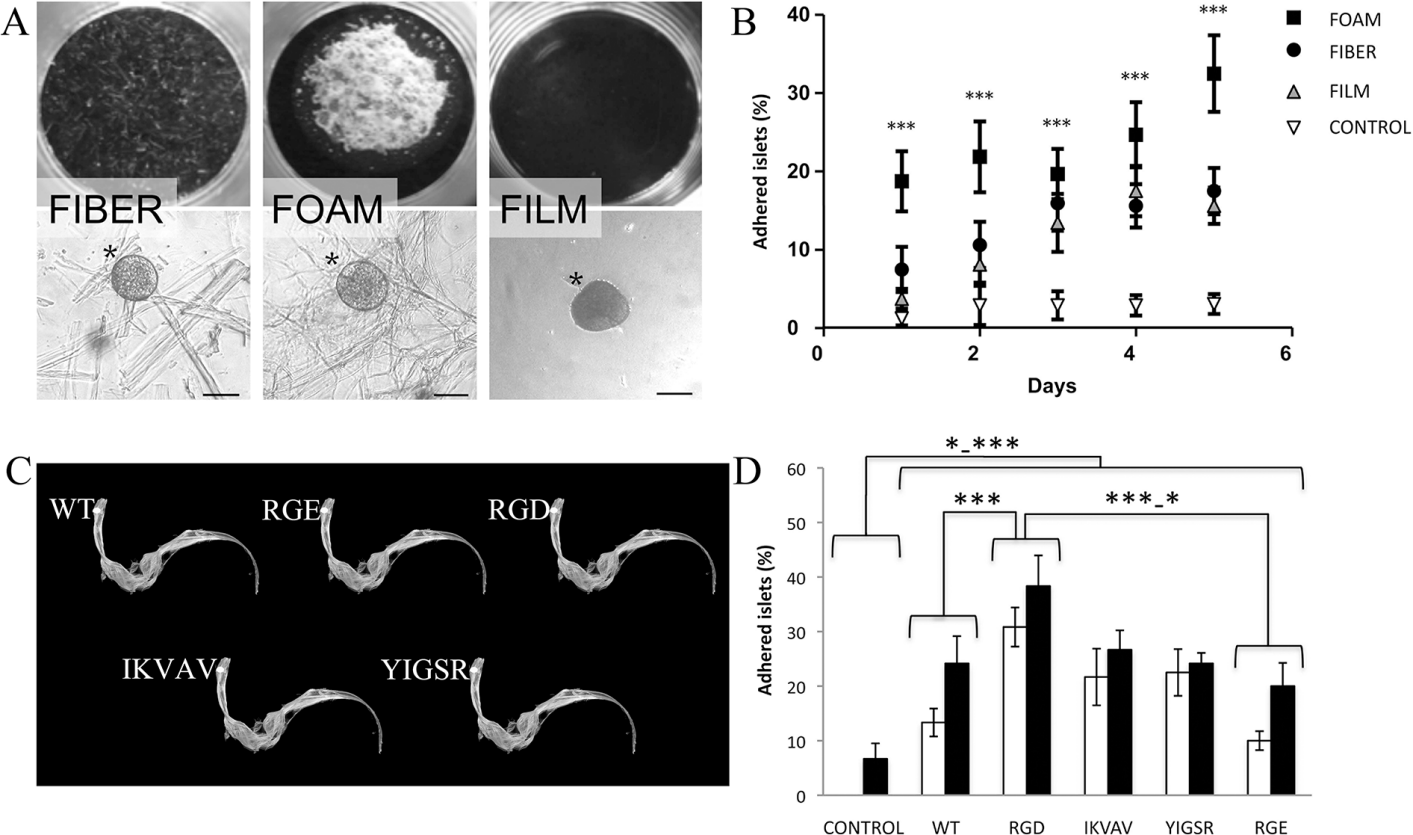
Mouse pancreatic islets adhere to silk matrices, with a preference for foam with RGD. **(A)** Photographs of the various formats; fiber (left), foam (middle) and film (right) and micrographs together with adhered islet (lower panel, asterisks). Scale bars = 50μm. **(B)** Islet adherence after different time points (days). Islets adhered better to foam, fiber and film, compared to the control *(n = 16*, *in duplicates*, ***p<0.*001)*. **(C)** Schematic descriptions of the spider silk proteins used; wild type 4repCT (WT) and 4repCT with different cell binding motifs (RGD, IKVAV and YIGSR) incorporated N-terminus of 4repCT. The non-functional peptide RGE was incorporated as control for RGD. **(D)** Islet adherence at day 1–2 (white bars) and day 5 (black bars) on foam with different motifs (*n = 6*, *in duplicates*). Islets adhered better to all silks compared to control (*** p≤0.*001* at day 2, * *p≤0*.*05* for WT and RGE at day 5, and **p≤0.*001* for RGD, IKVAV and YIGSR at day 5). Islets adhered better to RGD compared to WT (*** *p≤0*.*001* for day 2 and day 5*)* and RGE (*** *p≤0*.*001* for day 2 and *p≤0.*05* for day 5).

Pancreatic islets are naturally surrounded by a supporting layer of ECM. We postulated that recombinant spider silk could be used as a temporary support after islet isolation. Spider silk proteins, based on 4repCT [14, 21], were purified and formulated into different formats such as fiber, foam and film (Fig 1A). Recombinant DNA technology allowed facile incorporation of cell binding peptides selected from laminin *i*.*e*. RGD, IKVAV and YIGSR (Fig 1C), in order to add cell adhesive functions to the matrices. The incorporated peptides did not affect the ability of 4repCT to form solid silk-like materials.

### *In vitro* maintenance of pancreatic mouse islets

Optimizations of the experimental setup and initial studies were done using mouse islets, due to their *in vitro* tractability.

#### Mouse islets preferably adhere to foam with RGD

First, we investigated which of the three silk-based formats; fiber, foam or film, that were most suitable for these studies. Mouse islets could adhere to all formats, whereas less than 7% of the islets adhered in the control wells (without matrices) (n = 16, Fig 1B). The preferred format for adhesion was found to be the foam structure, where more islets adhered compared to the other formats, and with an increase over time (Fig 1B). The following experiments were therefore performed on foam. Next we investigated if adherence could be improved with cell binding peptides incorporated into the matrices. The RGD peptide enhanced adherence significantly more compared to WT and RGE (n = 6, p≤0.05, Fig 1D).

#### Adhered islets have maintained function

**Fig 2:**
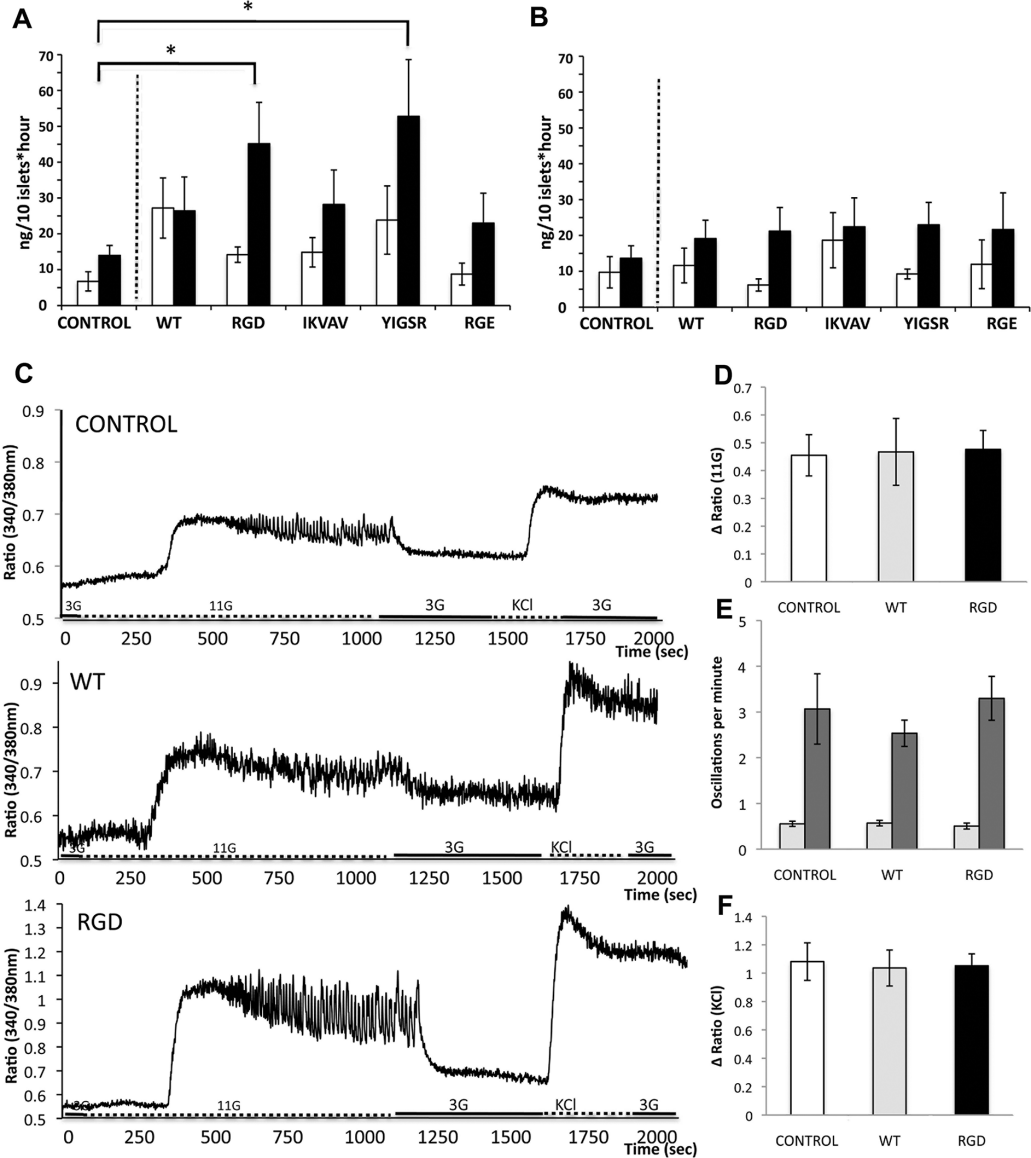
Mouse pancreatic islets are maintained functional when adhered to silk foam. Insulin release (ng/10 islets*hour) from adhered islets after 2 days **(A)** and 5 days **(B)** in culture on silk matrices (WT, RGD, IKVAV, YIGSR and RGE), compared to non-adhered islets in control wells. Insulin released after low glucose concentration (3mM) is shown in white bars and insulin released after stimulating glucose concentration (16.7mM) is shown in black bars. At day 2, adhered islets on RGD and YIGSR released more insulin per islet after glucose stimulation compared to control islets (*n = 6*, *triplicates* *p≤0.*05*). **(C)** Representative pattern of F_340_/F_380_ traces due to glucose and KCl induced changes in [Ca^2+^]_*i*_ in single mouse islets cultured for 1–2 days on WT, RGD or free floating. **(D)** Mean value of increase in ratio due to stimulation by 11 mM glucose. **(E)** Mean value of islet oscillations per minute for slow (light gray) and fast (dark gray) oscillations in [Ca^2+^]_*i*_ after high glucose stimuli. **(F)** Mean value of increase in ratio due to stimulation by 25 mM KCl. (*>17 islets from each condition*).

To validate that function is preserved when islets are adhered to the silk matrices, they were subjected to glucose stimulation at day 2 and 5 (n = 6, triplicates, Fig 2). At day 2, islets that were adhered to RGD and YIGSR foam released significantly more insulin per islet compared to control islets that were free floating. At day 5 the adhered islets released the same amount of insulin per islet as did the control islets, confirming that adherence to the silk matrices allows maintained function. These glucose-challenge tests were done on pools of islets, typically 3–10 islets per well, and then normalized and shown as insulin released per islet in Fig 2. When analyzing the free-floating control islets, there were several wells (17% at day 2 and 50% at day 5) where the pool of islets did not respond to increase in glucose concentration (thus a stimulation index below 1). However, there were no wells with RGD foam where adhered islets did not respond with increase in insulin release, indicating reduced variableness among the adhered islets.

Next, we therefore investigated the function of individual islets, by measuring concentration of cytoplasmic free calcium, ([Ca^2+^]_*i*_), after glucose and KCl stimulation (Fig 2C–2F). When islets are stimulated with high glucose concentration, the [Ca^2+^]_*i*_ increases and gives a subsequent oscillatory response [25]. Viable islets were selected after culturing for 1–2 days either in suspension or adhered to WT or RGD foam. Approximately 95% of the islets, no matter of culture condition, showed an increase in [Ca^2+^]_*i*_ due to high glucose stimulation, with similar amplitude (Fig 2D). A majority of the islets on RGD foam (85%) and control islets (79%) responded with [Ca^2+^]_*i*_ oscillations, thus in agreement with previous results of viable islets [28]. Slightly fewer islets on WT (62%) responded with oscillations, however with no differences in slow or fast oscillation pattern (Fig 2E). After return to basal glucose concentration, the oscillations stopped and the [Ca^2+^]_*i*_ decreased again. Subsequent addition with KCl gave an elevated influx of Ca^2+^, due to membrane depolarization, with no significant difference between the experimental islet groups (Fig 2F), confirming viability of the measured islets. Thus, if only viable islets were measured, the functionality was equal for adhered and free-floating islets.

#### Adhered islets show less necrosis

**Fig 3:**
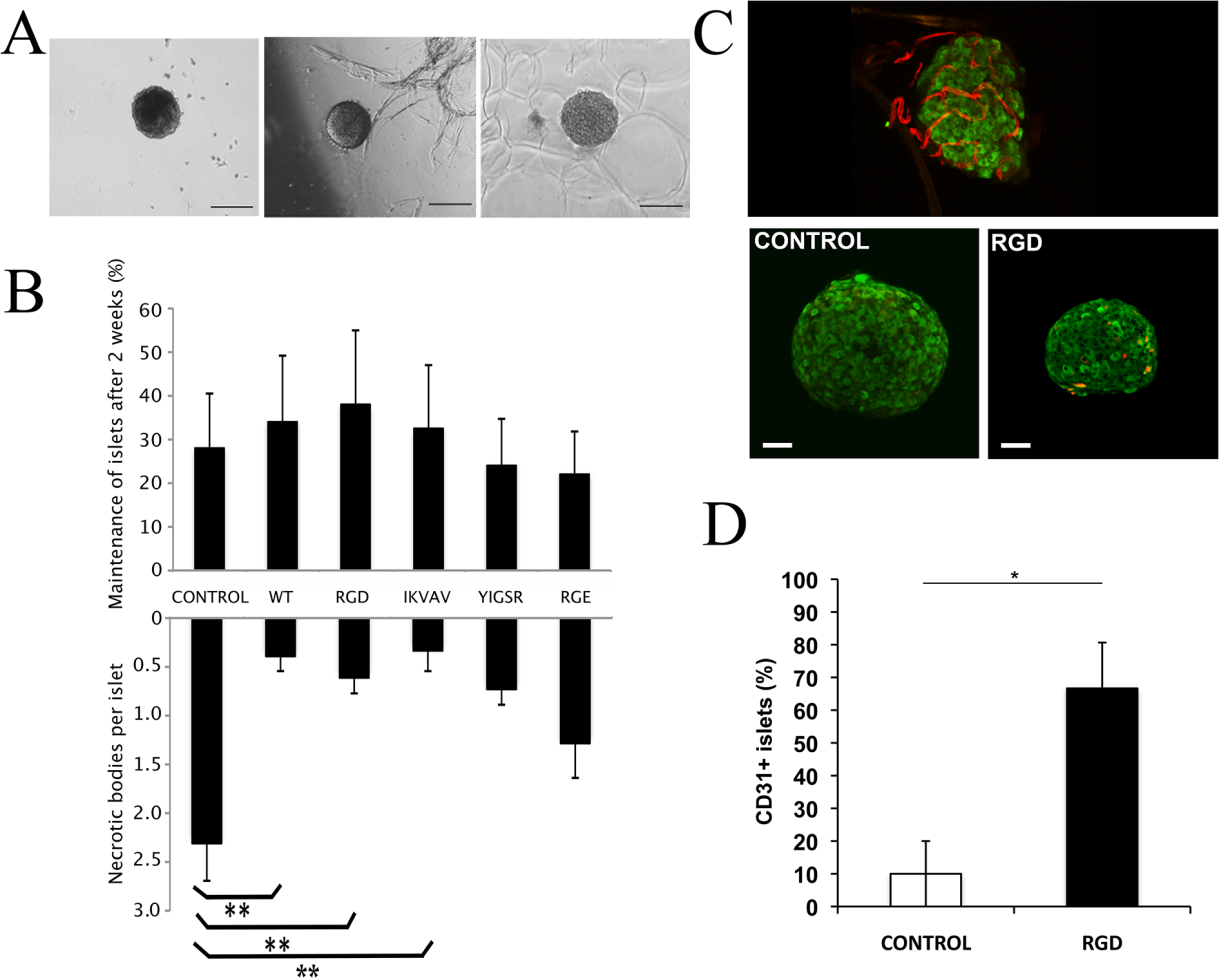
Adherence to silk matrices leads to improved *in vitro* islet maintenance with preserved endothelial cells. **(A)** Representative (*n = 5*) micrographs of islets after 2 weeks in control wells (left), on WT foam (middle) and on RGD foam (right). Scale bars = 50 μm. **(B)** Upper graph: Percentage of amount of islets with intact islet architecture after 2 weeks free floating in control wells or adhered to silk foam (WT, RGD, IKVAV, YIGSR and RGE). Lower graph: Mean value of necrotic bodies per islets after 2 weeks in control wells or on foam. Islets on WT, RGD and IKVAV foam showed less necrotic bodies compared to control (*n = 5*, *in duplicates* **p≤0.*01*). **(C)** Representative (*n = 3*) maximum intensity projection (MIP) micrographs of pancreatic mouse islets at the day of isolation (upper panel) and after 2 weeks in culture (lower panel). Insulin staining is shown in green and the endothelial marker CD31 is shown in red. Scale bars = 50 μm. **(D)** Percentage of islets with preserved endothelial cells (CD31-positive stained islets) after 2 weeks in culture (*data pooled from 3 experiments*, *in total 22 islets* *p≤0.*05)*.

We speculated that the observed variability in response to glucose concentration for islets after culture in suspension could be due to a decrease in viability. Indeed, after 2 weeks in culture, more mouse islets (38±17%) were maintained and kept their integrity on RGD foam compared to control islets (28±12%)(Fig 3B). Light-microscopy analysis showed that necrotic bodies had formed within the islets after 2 weeks in culture (Fig 3A). However, significantly less necrotic bodies were found in islets that were adhered to WT, RGD and IKVAV foam, compared to free floating islets (Fig 3B), possibly explaining the observed trend in islet maintenance.

#### Adhered islets show prolonged survival of endothelial cells

We speculated that the improved maintenance could be linked to a preserved islet architecture including supportive vasculature. Immediately after isolation, islets were found to be surrounded by a nice and well-spread vasculature (Fig 3C). After culture of islets for 2 weeks on RGD foam some preserved endothelial cells where still seen, whereas barely any cells positive for the endothelial cell marker CD31 were found in the control islets (Fig 3C and 3D).

Next, we wanted to investigate if culture of islets on RGD foam in this way could improve their performance upon transplantation, in terms of viability and engraftment.

### *In vivo* imaging and analysis of transplanted grafts

Transplantation into the anterior chamber of the eye has previously been reported as a suitable method for evaluation of islet cell biology [27]. Herein, we used the open window offered by the eye for evaluation of viability and engraftment of islets that had been cultured for 2 weeks either on RGD foam or free floating.

#### Foam matrices are preserved in the eye without causing side effects

In order to evaluate the *in vivo* compatibility of the silk material itself, foam matrices of WT and RGD silk were followed for 1 year post transplantation into the anterior chamber of the eye. The animals recovered well from anesthesia and showed little irritation caused by the transplants. During the first 2 weeks a wound healing process was observed at the incision area, which healed well with no visual signs of irritation or inflammation. Throughout the period, there were no visual signs of interfering cell infiltration or indication of inflammation process around the foam matrices. Both variants of foam (WT and RGD) were kept intact with the same shape throughout the whole year. Moreover, there were no signs of degraded foam products in the eye neither acute nor after longer time post transplantation. Most of the matrices were found on the very same spot throughout the period, suggesting at least partial engraftment onto the iris (data not shown).

#### Islets cultured on RGD foam show improved engraftment

**Fig 4:**
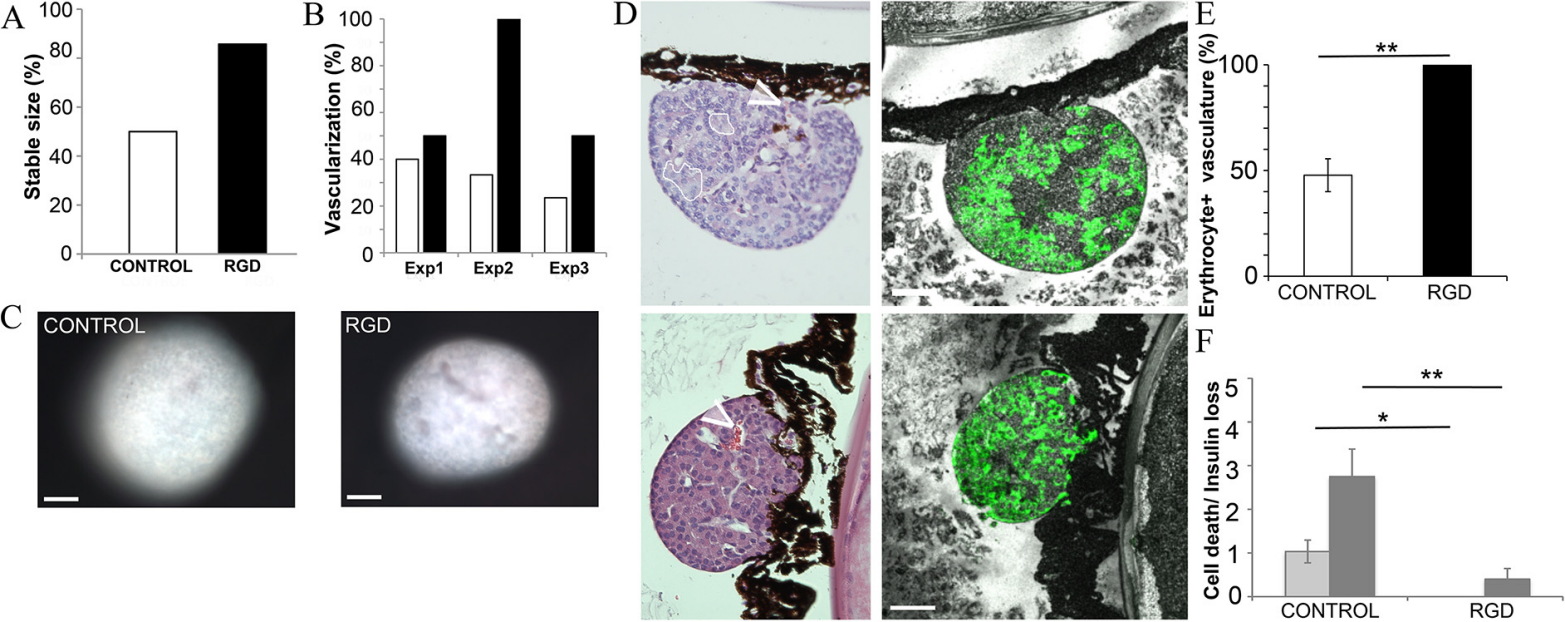
Islets maintained on RGD foam show improved *in vivo* survival with vascularization. **(A)** Percentage of islets that showed a stable or positive increase in size over the 4 weeks period in the eye. *(5 mice*, *1–6 islets per eye)*. **(B)** Percentage of control islets (white bars) and islets from RGD foam (black bars) that showed clear vascularization by transmitted light imaging 4 weeks post transplantation. (*n = 3 separate transplantation experiments*, *12 recipient mice in total*, *1–6 islets per eye*). **(C)** Representative (*n = 3*) bright field MIP micrographs of control islet (left) and islet from RGD foam (right) during *in vivo* imaging of the eye, where vasculature can be seen as grey areas. Scale bars = 50 μm. **(D)** Morphology by H/E (left panel) and insulin (green, right panel) staining of eye sections showing representative (*n = 3*) control islet (upper graphs) and islet from RGD foam (lower graphs). Vasculature was seen in islets from both culture conditions (white arrowhead) although vessels with erythrocytes were more common in islets from RGD foam. Areas of visual cell death were sometimes present in control islet (white lined circle). Scale bars = 50 μm. **(E)** Vasculature with erythrocytes were found within all (100%) of the islets from RGD foam (black bar), compared to in only 50% of the control islets (white bar)(mean % of islets found per eye ± SEM). (*n = 3*, *5–10 islets per eye* **p≤0.*01*). **(F)** Cell death (light grey) and insulin negative cells (dark grey) was seen to a higher degree in control islets compared to islets cultured on RGD foam (mean per islets found per eye ± SEM). (*n = 3*, 5–10 islets per eye *p≤0.*05*, **p≤0.*01*).

Since the silk matrices proved suitable *in vivo*, we continued with transplantation studies using islets cultured on RGD foam. During the 4 weeks transplanted into the eye, 86% of the islets that had previously been cultured on RGD foam maintained or increased their size, whereas only 50% of control islets, that had been cultured free floating without foam, were stable in size (Fig 4A). Similarly, the percent of islets showing clear vascularization after 4 weeks was higher for islets cultured on RGD foam before being transplanted into the eyes (Fig 4B and 4C), which correlate well with the amount of preserved endothelial cells (CD31 positive staining) on the islets after 2 weeks *in vitro* (Fig 3C and 3D).

At the endpoint, the animals were sacrificed and their eyes with grafts taken for histology evaluation. Sliced eye sections were H/E stained in order to evaluate islets morphology and incorporation into the host. Both islets from RGD foam and control islets were found attached onto the host iris with good integrity and morphological appearance (Fig 4D).

Intra islet capillaries were evaluated for erythrocyte content as an additional proof of revascularization. All of the islets supported by RGD foam had vasculature with erythrocytes present, whereas only half of the control islet had erythrocytes within their capillaries (Fig 4E).

Cell death was morphologically evaluated in the H/E stained sections of transplanted islets and graded 0–5, where 0 means no visual cell death and 5 means a high degree of cell death. Within control islets some areas with cell death were seen (1.03±0.26), whereas no visual cell death (0) could be observed within islet previously cultured on RGD foam (Fig 4F).

All transplanted islets showed indication of functionality by insulin positive cells within the core of the islets (Fig 4D). However, substantial areas without insulin positive cells were seen in some islets. After subtraction of areas corresponding to vasculature, the presence of insulin negative areas was graded from 0 to 5, where 0 means no insulin negative endocrine cells and 5 means high degree of insulin loss. Within control islets the insulin negative areas were more pronounced than in islets that had been maintained on RGD foam (Fig 4F).

### *In vitro* maintenance of human islets

As the recombinant silk foam seemed suitable for *in vitro* maintenance as well as *in vivo* engraftment of mouse islets, further studies were performed using human islets.

#### Human islets adhere with maintained function

**Fig 5:**
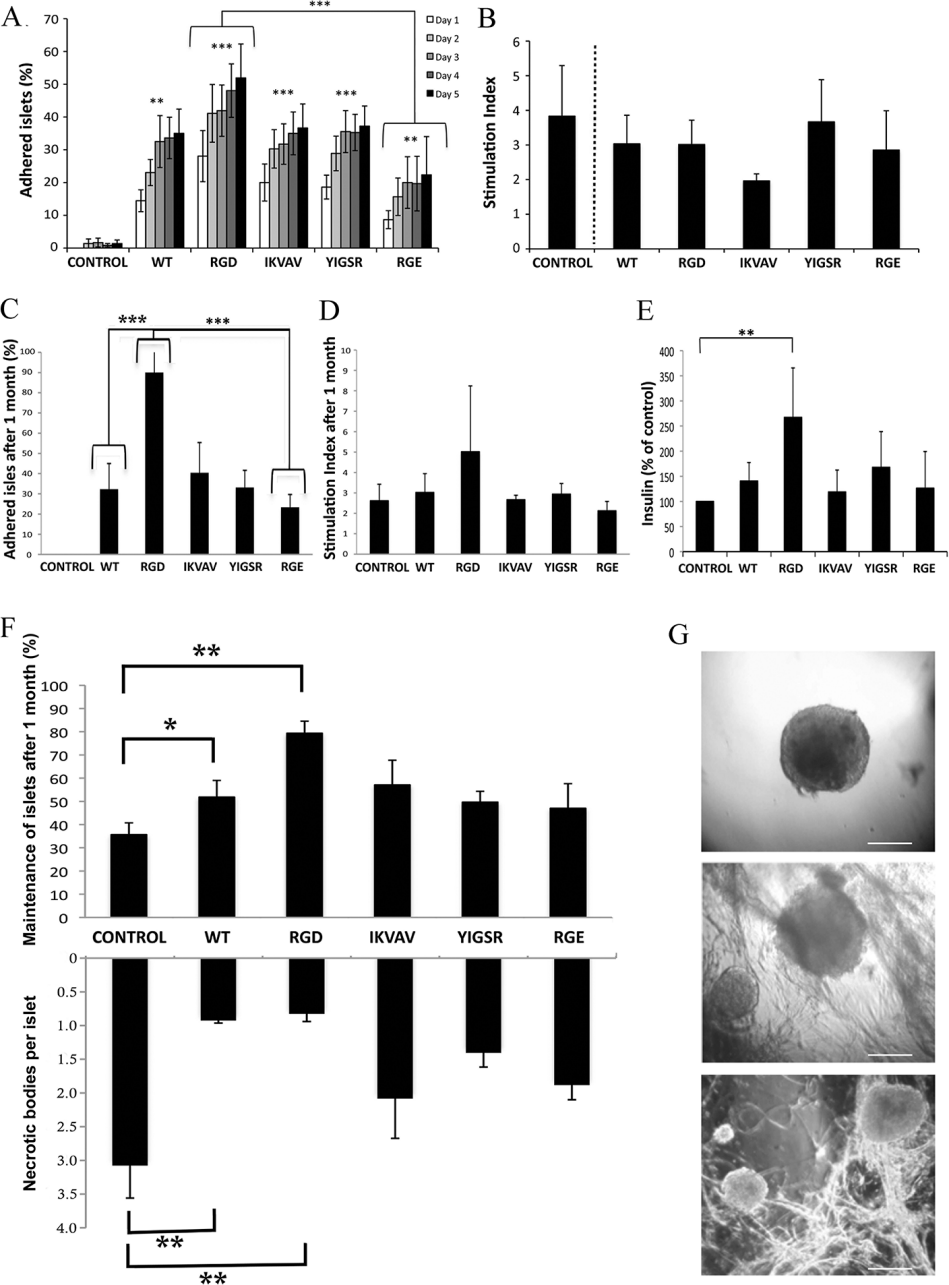
Human pancreatic islets adhere to silk matrices with maintained *in vitro* function for 3 months. (A) Islet adherence to silk matrices with various motifs (WT, RGD, IKVAV, YIGSR and RGE) after 1 to 5 days in culture. Islets adhered better to all silks compared to control (*n = 6*, *triplicates* **p≤0.*01*, ***p≤0.*001*). More islets adhered to RGD foam compared to RGE (***p≤ 0.*001)*. **(B)** Stimulation index of adhered human islets after cultured for 5 days on foam compared to non-adhered islets in control well. (*n = 6*, *triplicates)* **(C)** Islet adherence after 1 month of culture. No adhered islets were observed in the control wells. Islets on RGD foam showed improved adherence compared to WT and RGE (*n = 4*, *in triplicates* ***p≤0.*001*). **(D)** Islets remaining after 1 month in culture had maintained function of insulin release. The stimulation index of islets in control wells (non-adhered) and adhered to foam were all above 2 (*n = 4*, *in triplicates*). **(E)** Insulin produced after stimulation of all remaining islets within a well after 1 month of culture. Islets cultured on RGD foam showed increased insulin release compared to control islets (*n = 4*, *in triplicates*, **p≤0.*01)*. **(F)** Upper graph: Percentage of islets with intact islet architecture after 1 month in control wells or on foam. Islets were better maintained when cultured on WT and RGD compared to the islets in the control wells (*n = 5*, *triplicates*, *p≤0.*05*, **p≤0.*01*). Lower graph: Mean value of necrotic bodies per islet after 1–3 months in control wells or on foam. Islets adhered to WT and RGD foam showed less necrosis compared to control islets (*n = 4*, *triplicates* **p≤0.*01*). **(G)** Representative micrographs of islets after 3 months in control wells (top), wells with WT foam (middle) and RGD foam (bottom). Scale bars = 100 μm.

Human islets adhered to the silk foam to a larger extent than the mouse islets did, with hardly any adhered islets in the control well. During the first five days, an increase in adherence was observed, with most islets (50±10%) adhered to foam with RGD (p≤0.001) (Fig 5A). A comparison between RGD and RGE showed adherent specificity to the cell-binding motif RGD.

To confirm that the human islets were still functional after 5 days adhered to the silks, they were subjected to glucose stimulation. Adhered and non-adhered control islets retained their ability to respond to high glucose, yielding a similar stimulation index (all above 1) (Fig 5B).

#### A majority of the islets are adhered with maintained function after long term culture

After 1 month in culture almost all islets within the wells (90±14%) had adhered to the RGD foam, while no adhered islets were found in the control wells (Fig 5C). The insulin secretion assay was performed again and confirmed maintained function of the remaining islets (Fig 5D and 5E). The adhered islets could still respond to increased glucose concentration with a stimulation index above 2 for islets adhered to matrices, highest for islets on RGD foam (Fig 5D).

#### Adhered islets have intact architecture and less necrosis

After long-term culture there were more intact islets preserved in wells with silk, and especially on RGD-foam (Fig 5F). Therefore, the total amount of insulin released by islets within these wells was significantly increased (Fig 5E). Comparison of the amount of insulin produced with the number of viable islets present showed that the function of individual adhered islets were maintained, but not enhanced, during long-term culture. However, since adherence to the silk matrices increased the amount of maintained and functional islets, this lead to a significant increase in total insulin production (Fig 5E).

As observed for mouse islets, those few human islets that were still found in the control wells had significantly more necrotic bodies (Fig 5F and 5G), indicating that necrosis was the major cause of islet disruption.

#### Islets form sprouts and new islet-like clusters after long term culture on RGD foam

**Fig 6:**
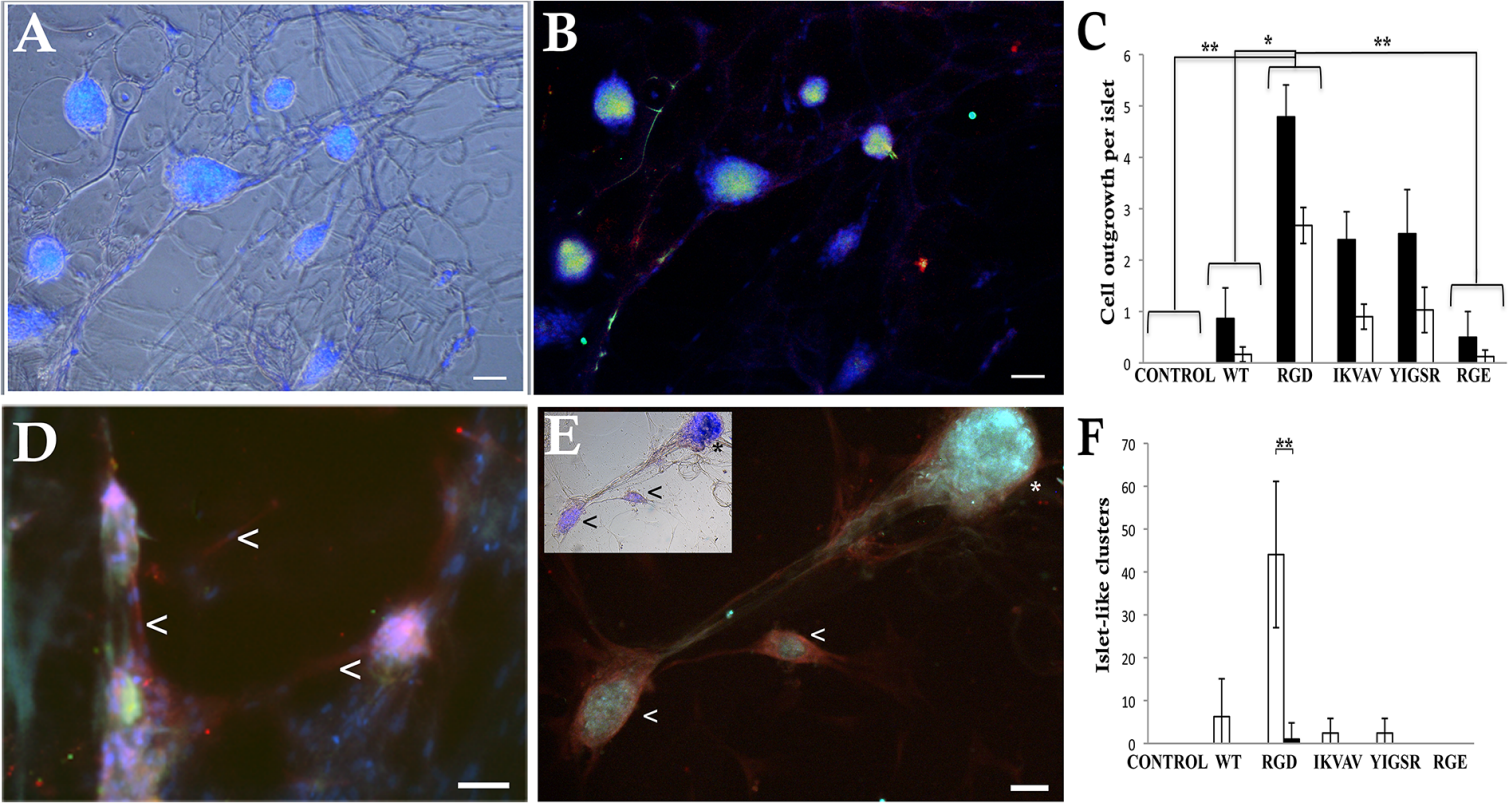
Human pancreatic islets are well established with connecting cell growth following the shapes of the silk matrices. **(A)** Human islets adhered preeminent to the RGD foam (bright field) and were connected in a network by cell outgrowth following the foam structure (cell nuclei seen in blue). **(B)** The islets maintained viable and produced insulin (green) after culture for 4 weeks (cell nuclei seen in blue). **(C)** Mean value of number of ordinary cell outgrowths (black) and angiogenic sprouts (white) found per human islets cultured for 4–12 weeks on different matrices. Islets adhered on RGD foam showed more cell outgrowth and sprouts compared to control, WT and RGE (*n = 4*, *triplicates* *p≤0.*05 and* **p≤0.*01*). **(D)** Example of angiogenic sprouting (white arrowhead). Endothelial cells (red) emanating from one insulin positive (green) islet and connecting to another (cell nuclei seen in blue). **(E)** Except for maintained islets (asterisks) some newly formed insulin-positive (green) islet-like clusters (arrow head) were found, and these were also connected by sprouting endothelial cells (red) guided by the foam structure. Upper left: the same picture in bright field (cell nuclei seen in blue). **(F)** Mean value of islet-like clusters after 4–12 weeks from young (white bars) and old (black bars) islet donors. Most islet-like clusters were found on the RGD foam. Islets from young donors showed increased number of islet-like clusters compared to the old islet donors (*2 young donors*, *islet wells in triplicates and 3 old donors*, *islet wells in triplicates* **p≤0.*01)*. Scale bars = 50 μm.

Many of the adhered islets showed cell outgrowth along the foam matrices, still with preserved islets architecture (Fig 6A and 6B). Significant cell outgrowth was seen from islets of all human donors when cultured on the RGD foam, although cell outgrowth was commonly observed also from islets cultured on IKVAV and YIGSR (Fig 6C). Approximately 50% of the cell outgrowth stained positive for the endothelial cell marker CD31, indicating sprout formation (Fig 6C, 6D and 6E). Interestingly, newly formed islet-like clusters could also be found (Fig 6E and 6F). These clusters were attached to the matrices and showed positive staining for insulin. When comparing cluster formation with age of the islet donor, we found that it was mainly islets from younger donors (< 35 years) that had the ability to form islet-like clusters, and that this occurred particularly on the RGD foam (Fig 6F). Some of the cells between the newly formed islet-like clusters and the islets also stained positive for endothelial cell marker CD31, indicating angiogenic sprout formation (Fig 6E).

## Discussion

Several strategies to promote recovery and maintenance of isolated islets have been tried throughout the years. Complex ECM deposits isolated from carcinoma cell lines [29–33] or reconstituted basal membrane extracts [34] have been used as substrates for culturing of islets, and shown to give both positive and negative effects. The effect of individual ECM proteins have been investigated separately [8, 35–39], but when these proteins are used to coat plastic plates they often promote formations of cell monolayers from the islets, followed by loss of insulin secretion [32, 38, 40]. Therefore, attempts have been made to construct flexible 3D supports, *e*.*g*. encapsulations [41] [42], both from synthetic [43–45] and natural polymers [46–51], which generally have given positive effects. Herein, more islets adhered to the 3D foam than to the film or fibers. The silk-based foam allowed the islets to adhere without losing their architecture, since the flexible foam could adjust to the shape of the islets, which were often found within grooves of the foam structure.

Natural ECM proteins are tedious to produce synthetically and, except for collagen, they are generally difficult to process into 3D formats. To investigate the effects of natural cell-matrix interactions in 3D, selected cell binding peptides can be incorporated to a synthetic 3D matrix. MIN6 insulin-releasing cells exhibited reduced viability when encapsulated in photopolymerised PEG hydrogel, but regained increased viability after incorporation of laminin-derived peptides [43]. In a recent study by Davis *et al* [52] the addition of laminin or collagen to a silk fibroin hydrogel was shown to increase the *in vitro* functionality of mouse islets. Herein, three selected laminin-derived motifs, IKVAV, YIGSR and RGD, were covalently attached to spider silk. In this way, a defined material with no animal-derived components was constructed, which still allow ECM-like cell adhesion. The peptide IKVAV is naturally present in the laminin α1 chain while the peptide YIGSR is found in the β1 chain. The RGD peptide is found in several of the laminin α chains (although often cryptic), and also in *e*.*g*. collagen I, fibrinogen, fibronectin and vitronectin [53], and is the most widely characterized cell binding peptide. RGD is the peptide that showed most pronounced effects in these studies, both on adherence, viability, cell outgrowth and revascularization. RGD has previously been shown to interact with several integrin variants, *e*.*g*. αvβ3, αvβ5, α5β1 and, although with weaker interaction, with α3β1 [53–55]. Both αvβ3 and αvβ5 have previously been shown to regulate adhesion of islets, while other integrins, *e*.*g*. α3β1 and α6β1, are involved in regulation of insulin secretion [33, 56–58]. Human islets were more prone to adhere to the silk matrices compared to mouse islets, probably reflecting differences in islets architecture [59]. Human islets have a double basal membrane while in rodent islets the beta cells are gathered in the inner core and are in direct contact with the vascular basal membrane [39, 60]. There also seems to be species differences in the integrin pattern. The RGD-binding αv integrins have been found in human but not in rodent islets [10, 36, 61], which could explain the observed preference for adherence of human islets. Together with our observed differences in behavior between human and mouse islets, this emphasizes the necessity of studies of human material when the aim is translation into clinical applications.

Survival of islets is of great importance not only during transplantation but also during *in vitro* culture prior to transplantation. Herein, adherence to spider silk matrices, especially with RGD, increased maintenance of mouse islets with intact architecture from 28% to 38% after two weeks, and for human islets from 36% to 79% after one month. A possible explanation is that non-adherent islets have diminishing integrin expression, followed by a decrease in phenotypic characteristics leading to apoptosis [36], reflected by the observed increase in necrosis of free floating islets. However, it is difficult to distinguish between effects of binding and those related to the physical support given by the foam. The significantly improved islet mass, with maintained function, on silk with RGD points to the importance of receptor mediated adhesion, which is in line with previous findings that non-adhered cells are prone to undergo anoikis [61]. However, a large portion of beta cells are in the interior of the islet and do not have direct contact with the peripheral ECM, so any benefit they derive from matrix restoration must be transmitted indirectly *e*.*g*. by maintained architecture.

When islets are destined for transplantation, they should preferably be able to re-establish a functional niche during culture *in vitro*, and then continue proper engraftment *in vivo*. To avoid the need of disrupting cell-matrix interactions established *in vitro*, and to maintain physical support against hemodynamic shear stress, it is preferable that the matrix used for *in vitro* culture can also be transplanted together with the islets to promote cell attachment at the ectopic site. It is then important to choose a defined material with no animal-derived components. Herein, a recombinant silk matrix was first transplanted alone, which proved *in vivo* compatibility of the material at the intended site. Intra islet endothelial cells have previously been shown to diminish over time during *in vitro* culture [62]. However, immunostaining confirmed that endothelial cells were present in islets that had been maintained on RGD foam instead of free floating. These islets were also better engrafted with erythrocyte-containing vasculature and maintained or even increased islet size upon transplantation, probably due to that the support provided by the foam helped to retain endothelial cells within the islets. A decreased islet size indicates cell death which could be due to slow revascularization process and thereby also ischemia. The control islets showed a less stable size, fewer erythrocyte-contained capillaries and more areas of cell death.

During longer culture times of human islets, cell outgrowth resembling sprout formation could be seen, especially on RGD foam. The islet structure and morphology was not affected by the cell outgrowth, rather the outgrowing cells were found along the foam structures, connecting the islets with each other. In half of the cell outgrowths, endothelial cells were found emanating from the islets, and these sprouts indicate that the matrices promote angiogenesis. The integrin αvβ3, that mediate attachment to RGD sequences, is up regulated in endothelial cells of angiogenic vessels [63]. Islets are complex tissues with multiple interspaced cell types, *e*.*g*. endothelial cells, that are co-isolated even in the purest islet preparations [64]. This may be beneficial for engraftment and long-term maintenance since the beta-cells themselves do not form any ECM [17] but are dependent on surrounding endothelial cells [10] to produce basal membrane and vascular networks. The RGD-functionalized silk matrices seem thus to be beneficial due to their ability to maintain islet vasculature and act as a supporting matrix to the co-isolated endothelial cells.

Interestingly, human islets from donors under 35 years of age had the capacity to form insulin-positive islet-like clusters after long-term culture on the RGD foam. Sprouts, connecting the islet-like clusters with islets, could also be seen. Proliferation of beta cells from young donors has previously been reported, although at early time points only on laminin 411 and 511 [65]. Possibly, long-term culture of islets on RGD foam promotes the establishment of endothelial cells that start to produce suitable ECM proteins (*e*.*g*. laminin 411 and 511) and/or growth factors. Further studies of islet cells and their interactions with these functionalized matrices will likely provide valuable insights into the progress of islet neogenesis and vascularization.

## Conclusions

We have evaluated the usage of silk-based supports, functionalized with selected cell binding motifs, to re-establish an environment that allows maintenance of pancreatic islets after isolation and improves engraftment upon transplantation. Application of islets from human donors onto these functionalized silk matrices improved their long-term maintenance with intact architecture and function. The islets were able to establish cell-matrix contacts and formed new islet-like clusters that were connected via vessel-like structures along the matrices. The described procedure may facilitate both research use and clinical application of viable islets and islet-like clusters. Importantly, a natural-like *in vitro* model for established islets, as described herein, could be useful for screening of potential therapeutic treatments as well as for development of novel transplantation strategies.

## Supporting Information

S1 Fig Evaluation of necrosis after *in vitro* maintenance of mouse and human islets. During bright field light microscopy imaging of islets the necrotic areas can be seen as dark spots, denoted necrotic bodies. The amount of necrotic bodies was typically between 0 and 5 within each islet. **A)** Representative images of mouse islets after 2 weeks of culture as free-floating (left), on WT foam (middle) or RGD foam (right), including numbers of necrotic bodies from evaluation during live microscopy. **B)** Representative micrographs of human islets after 1 month of culture as free-floating (upper), on WT foam (middle) or RGD foam (lower), including numbers of necrotic bodies from evaluation during live microscopy.
(TIF)
