# Supplementary figures and images for: Pancreatic Islet Survival and Engraftment Is Promoted by Culture on Functionalized Spider Silk Matrices
Source: PLoS One. 2015 Jun 19;10(6):e0130169. doi: 10.1371/journal.pone.0130169 (PMC4474965; doi:10.1371/journal.pone.0130169)

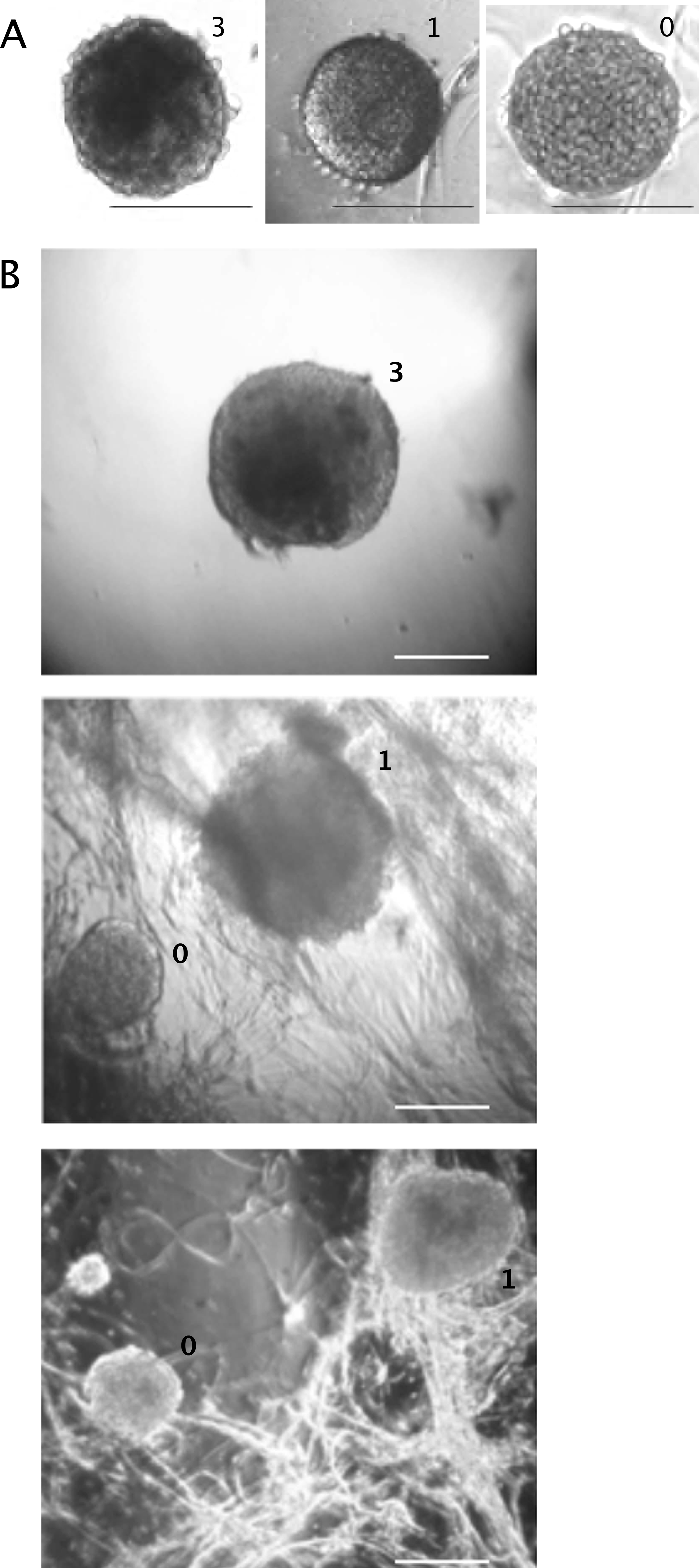

Supplement: S1 Fig — During bright field light microscopy imaging of islets the necrotic areas can be seen as dark spots, denoted necrotic bodies. The amount of necrotic bodies was typically between 0 and 5 within each islet. A) Representative images of mouse islets after 2 weeks of culture as free-floating (left), on WT foam (middle) or RGD foam (right), including numbers of necrotic bodies from evaluation during live microscopy. B) Representative micrographs of human islets after 1 month of culture as free-floating (upper), on WT foam (middle) or RGD foam (lower), including numbers of necrotic bodies from evaluation during live microscopy. (TIF) [file pone.0130169.s001.tif]
